# Wild Bird Influenza Survey, Canada, 2005

**DOI:** 10.3201/eid1401.061562

**Published:** 2008-01

**Authors:** E. Jane Parmley, Nathalie Bastien, Timothy F. Booth, Victoria Bowes, Peter A. Buck, Andre Breault, Dale Caswell, Pierre-Yves Daoust, J. Chris Davies, Seyyed Mehdy Elahi, Madeleine Fortin, Fred Kibenge, Robin King, Yan Li, Norman North, Davor Ojkic, John Pasick, Sydney Paul Pryor, John Robinson, Jean Rodrigue, Hugh Whitney, Patrick Zimmer, Frederick A. Leighton

**Affiliations:** * Centre for Coastal Health, Nanaimo, British Columbia, Canada; † Canadian Cooperative Wildlife Health Centre, Nanaimo, British Columbia, Canada; ‡ Public Health Agency of Canada, Winnipeg, Manitoba, Canada; § British Columbia Ministry of Agriculture and Lands, Abbotsford, British Columbia, Canada; ¶ Public Health Agency of Canada, Ottawa, Ontario, Canada; # Canadian Wildlife Service, Delta, British Columbia, Canada; ** Canadian Wildlife Service, Winnipeg, Manitoba, Canada; †† University of Prince Edward Island, Charlottetown, Prince Edward Island, Canada; ‡‡ Canadian Cooperative Wildlife Center, Charlottetown, Prince Edward Island, Canada; §§ Ontario Ministry of Natural Resources, Peterborough, Ontario, Canada; ¶¶ University of Montréal, St.-Hyacinthe, Québec, Canada; ## Ministère de l'Agriculture, des Pêcheries et de l'Alimentation du Québec, Québec, Québec, Canada; *** Alberta Agriculture and Food, Edmonton, Alberta, Canada; ††† Canadian Wildlife Service, London, Ontario, Canada; ‡‡‡ University of Guelph, Guelph, Ontario, Canada; §§§ Canadian Food Inspection Agency, Winnipeg, Manitoba, Canada; ¶¶¶ Canadian Wildlife Service, Edmonton, Alberta, Canada; ### Canadian Wildlife Service, Ste.-Foy, Québec, Canada; **** Newfoundland and Labrador Department of Natural Resources, St. John’s, Newfoundland and Labrador, Canada; †††† Canadian Cooperative Wildlife Centre, Saskatoon, Saskatchewan, Canada

**Keywords:** Avian influenza, birds, Canada, surveys, ducks, PCR, surveillance, dispatch

## Abstract

Of 4,268 wild ducks sampled in Canada in 2005, real-time reverse transcriptase–PCR detected influenza A matrix protein (M1) gene sequence in 37% and H5 gene sequence in 5%. Mallards accounted for 61% of samples, 73% of M1-positive ducks, and 90% of H5-positive ducks. Ducks hatched in 2005 accounted for 80% of the sample.

To provide baseline information about the strains and distribution of influenza viruses in Canadian wild ducks and to respond to the emergence of highly pathogenic avian influenza (HPAI) type H5N1 in Asia, Europe, and Africa, Canada’s Interagency Wild Bird Influenza Survey was initiated in July 2005. The goals of the survey were to identify avian influenza viruses in wild ducks in Canada and to detect HPAI strains early (*1*). We report the results of real-time reverse transcriptase–PCR (RRT-PCR) analysis.

## The Study

Single cloacal swabs were collected from apparently healthy ducks at 56 sites within 6 geographic regions: British Columbia, Alberta, Manitoba, Ontario, Québec, and the Atlantic provinces (New Brunswick, Nova Scotia, Prince Edward Island, and Newfoundland and Labrador) (Figure). The target number of samples for each region was 800: 500 from mallard ducks (*Anas platyrhynchos*) and 300 from other duck species. Ducks were trapped and handled as part of annual duck banding carried out by the Canadian Wildlife Service and its associates. They were either caught in baited traps or netted from air boats. In all regions, duck banders were asked to preferentially sample birds hatched in 2005 to maximize virus detection within the sample (*2*–*4*).

**Figure:**
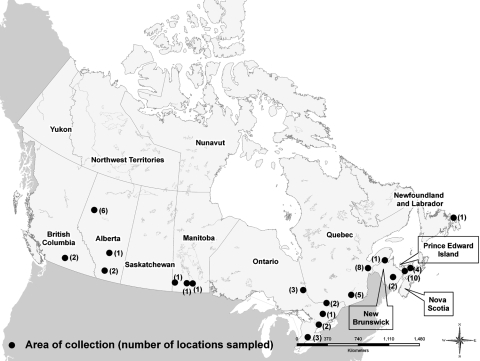
Live bird survey sample site locations.

Swabs were immediately placed in virus transport medium, refrigerated for up to 3 days, and then frozen at <–20°C until tested. The transport medium comprised Hanks balanced salt solution supplemented with 10% glycerol, 200 U/mL penicillin, 200 μg/mL streptomycin, 100 U/mL polymyxin B sulfate, 250 μg/mL gentamicin, and 50 U/mL nystatin.

All samples were tested at regional veterinary diagnostic laboratories within Canada's Influenza Virus Laboratory Network by RRT-PCR, which targets a conserved region of the M1 gene within influenza A segment 7. If the M1 gene sequence was detected, RRT-PCR for H5 and H7 hemagglutinin gene segments was performed. All laboratories followed uniform procedures (*5*) with positive and negative controls, and quality assurance was provided by the National Centre for Foreign Animal Disease (Canadian Food Inspection Agency).

All field and laboratory data were entered directly into a national database developed and maintained by the Canadian Cooperative Wildlife Health Centre. Duplicate records and any sample records missing RRT-PCR results or field collection data (species, age, sex, location) were removed from this analysis. Data were exported to Microsoft Excel 2003 (Microsoft Corp., Redmond, WA, USA) and prepared for analysis. Statistical analysis was performed with Epi-Info version 3.3.2 (Centers for Disease Control and Prevention, Atlanta, GA, USA) and SAS version 9.1 (SAS Institute Inc., Cary, NC, USA). Maps were generated in ArcGIS 9.0–ArcMap version 9.1 (ESRI, Redlands, CA, USA).

To assess differences in M1 and H5 virus detection by age class and species, main effects logistic regression models were constructed by using a manual stepwise procedure. Age class, species, sampling area, and sex were included in the model. Age class was categorized into 2005 hatch-year birds and birds from another hatch year. Species was categorized into mallard (mallard and mallard–American black duck [*A. rubripes*] hybrids), other dabbling duck species, diving ducks, and other tribes (Table 1). All variables were assessed for potential confounding; variables that changed estimates by >20% were left in the main effects models as confounders (*6*).

**Table 1:** Duck species sampled in 2005 and RRT-PCR results, Canada*

Common name	Taxonomic name	No. sampled	No. M1-positive (%)	No. H5-positive (%)
Mallard†	*Anas platyrhynchos*	2,600	1148 (44)	187 (7)
American black duck†	*A. rubripes*	293	99 (34)	2 (1%)
American wigeon†	*A. americana*	101	33 (33)	0
Blue-winged teal†	*A. discors*	431	105 (24)	2
Cinnamon teal†	*A. cyanoptera*	4	1 (25)	0
Gadwall†	*A. strepera*	36	1 (3)	0
Green-winged teal†	*A. crecca*	224	52 (23)	5 (2)
Northern pintail†	*A. acuta*	131	26 (20)	4 (3)
Northern shoveler†	*A. clypeata*	4	0	0
Wood duck‡	*Aix sponsa*	104	71 (68)	2 (2)
Common goldeneye§	*Bucephala clangula*	18	2 (11)	0
Canvasback§	*Aythya valisineria*	19	0	0
Hooded merganser§	*Lophodytes cucullatus*	26	8 (31)	0
Lesser scaup§	*Aythya affinis*	1	0	0
Redhead§	*Aythya americana*	223	18 (8)	6 (3)
Ring-necked duck§	*Aythya collaris*	51	8 (16)	0
Ruddy duck¶	*Oxyura jamaicensis*	2	0	0

*RRT-PCR, real-time reverse transcriptase–PCR. †Dabbling duck (Tribe Anatini). ‡Perching duck (Tribe Cairinini). §Diving duck (Tribe Aythini and Tribe Mergini). ¶Ruddy duck (Tribe Oxyurini).

## Conclusions

A total of 4,268 valid sample records were available for this analysis; 37% (1,572) of ducks were M1-positive and 5% (208) were H5-positive by RRT-PCR. No samples tested positive for the H7 gene sequence. Sampled areas varied considerably in the proportion of M1-positive samples, ranging from 63% (348/556) in southern Quebec to 9% (22/254) in southern Alberta (Table 2). Three percent (138) of samples were collected in July, 83% in August (3,539), 11% (454) in September, 1% (59) in October, none in November, and 2% (73) in December. Of all samples, 80% (3,401/4,268) were collected from ducks hatched in 2005. Hatch-year ducks accounted for 88% (1,388/1,572) of M1-positive and 99% (205/208) of H5-positive samples. Of all ducks sampled, 90% (3,824/4,268) were dabbling ducks (tribe Anatini) (Table 1). Mallards (including mallard–American black duck hybrids) accounted for 61% (2,600/4,268) of all ducks sampled, 73% (1,148/1,572) of all M1-positive samples, and 90% (187/208) of all H5-positive samples. The duck species with the highest proportion of M1-positive samples was the wood duck (*Aix sponsa*) (68%, 71/104).

**Table 2:** Sample sizes and RRT-PCR results for each sampling area included in the 2005 wild duck survey for influenza A viruses, Canada*

Region/area (no. sites sampled)	No. valid samples	No. M1 RRT-PCR positive (%)	No. H5 RRT-PCR positive (%)†
British Columbia interior (2)	640	351 (55)	161 (25)
Alberta			
Northern (6)	260	30 (12)	0
Central (1)	262	25 (10)	0
Southern (2)	254	22 (9)	0
Manitoba			
Western (1)	175	21 (12)	0
Central (1)	174	25 (10)	3 (2)
Eastern (1)	175	48 (27)	1 (1)
Ontario			
Southwestern (3)	269	135 (50)	3 (1)
Southcentral (2)	23	11 (49)	1 (4)
Central (1)	48	24 (50)	0
Eastern (2)	144	103 (72)	3 (2)
Northern (3)	284	66 (23)	2 (1)
Québec			
Southern (5)	556	348 (63)	28 (5)
Eastern (8)	221	32 (14)	0
Atlantic provinces‡			
Northern New Brunswick (1)	15	6 (40)	0
Central New Brunswick (2)	20	9 (20)	0
New Brunswick–Nova Scotia border (10)	646	290 (45)	6 (1)
Prince Edward Island (4)	21	13 (62)	0
Newfoundland and Labrador (1)	73	8 (11)	0
Total	4,268§	1,572 (37)	208 (5)

*RRT-PCR, real-time reverse transcriptase–PCR. †All M1-positive samples were subsequently tested for H5. ‡New Brunswick, Nova Scotia, Prince Edward Island, and Newfoundland and Labrador. §The sampling area of 8 ducks was not recorded (3 from Alberta, 1 from Ontario, 4 from Québec).

Age, area sampled, and species were included in the final logistic regression model for M1 RRT-PCR test results. Age, sex, and species were included in the final model for H5 RRT-PCR results. Mallards were more likely to be M1-positive than other dabbling ducks or diving ducks but not more likely to be M1-positive than other duck tribes (Cairinini and Oxyurini). Mallards were more likely to be H5-positive than other dabbling ducks, diving ducks, or other duck tribes. Hatch-year ducks were 1.7 times more likely to be M1-positive and 13 times more likely to be H5-positive than older ducks. Male ducks were 1.4 times more likely to test positive by H5 RRT-PCR than female ducks.

These data provided a snapshot of the frequency and distribution of influenza A viruses in wild ducks across southern Canada in 2005. This unique nationwide snapshot showed wide variation in detection of M1 and H5 gene sequences among 6 regions that were broadly representative of the northern terminus of duck migration corridors in North America (*7*). Previous studies have focused on smaller subregions of Canada sampled once or across multiple years (*4*,*8*–*10*). Direct comparisons with previous studies should be made cautiously. Considerable variation exists across studies in the age and species of birds sampled and seasonality of sampling. All of these variables may affect reported prevalence of infection with influenza A viruses. Also, most previously published results relied on the cultivation of viable virus as the detection method rather than on RRT-PCR applied directly to cloacal swabs.

Because samples for this survey were secured from preestablished duck-banding operations, and young healthy ducks, particularly mallards, were targeted to maximize virus recovery (*1*,*11*,*12*), these data may not represent true infection prevalence in the sampled duck populations. Despite these biases, avian influenza viruses were common in wild ducks across Canada in the summer and fall of 2005. Infection rates detected by RRT-PCR among the 6 regions of Canada were similar in scale and degree of variation to rates reported from the Alberta region, which were measured over 8 consecutive years from 1976 through 1983 (*4*).
